# Two Adhesive Sites Can Enhance the Knotting Probability of DNA

**DOI:** 10.1371/journal.pone.0132132

**Published:** 2015-07-02

**Authors:** Saeed Najafi, Raffaello Potestio

**Affiliations:** Max Planck Institute for Polymer Research, Ackermannweg 10, 55128 Mainz, Germany; Weizmann Institute of Science, ISRAEL

## Abstract

Self-entanglement, or knotting, is entropically favored in long polymers. Relatively short polymers such as proteins can knot as well, but in this case the entanglement is mainly driven by fine-tuned, sequence-specific interactions. The relation between the sequence of a long polymer and its topological state is here investigated by means of a coarse-grained model of DNA. We demonstrate that the introduction of two adhesive regions along the sequence of a self-avoiding chain substantially increases the probability of forming a knot.

## Introduction

In the last few decades, concepts from Topology have increasingly gained ground in the study of biopolymers, most notably in the case of proteins [1–6] and DNA [7–14]. These molecules, in fact, can undergo the same fate of an everyday piece of rope: they can be knotted. Characterized by a wealth of three-dimensional conformations and traditionally described in structural terms, biopolymers have demonstrated to entail a similarly rich variety of topological features, which largely affect their behavior [15, 16]. Knotted protein folds, for example, have long been associated only with folding errors [17, 18], whether *in vivo* or *in silico*. To the date of writing, the KnotProt [19] database lists about 800 protein entries with a proper knot (i.e. no slipknots), the functional relevance of which is often under debate. On the contrary, a long polymer chain such as DNA, with a much smaller degree of sequence-dependence with respect to a protein, is expected to be knotted with high probability [20–22]. It is hence not surprising to find experimental as well as numerical evidence of topological entanglement in DNA strands confined in viral capsids [9, 11]. On the other hand, it comes to much surprise that the 100 Mbp-long genetic material contained in a human chromosome is knot-free [23]. Recently, the same puzzling absence of knots has been observed also in RNA molecules [24]. The well established biology paradigm *sequence* → *structure* → *function* [25] is then enriched with topology, and it is of the greatest importance to understand the interplay between these four instances. To shed light on this conundrum, a body of work has been carried out especially by means of numerical simulations, with focus on different aspects of the problem, for example knotted protein folding [4, 5, 26–28], DNA [10–13, 23], knot-specific sequences in model polymers [29–31], and equilibrium properties of knotted chains [15, 16, 21, 22, 32–35].

In the present article, we focus our investigation on the relation between sequence and topology of DNA. Genetic material, in fact, is at the center of a broad range of topology-related biological and material science problems. The self-recognition of complementary sequences allows the formation of nontrivial two- and three-dimensional structures. This property lies at the core of genetic recombination, and enables the occurrence of secondary structure element formation in RNA macromolecules, as is in the case e.g. of ribosomes. The possibility to form selective, sequence-dependent bonds has been widely exploited in the field of structural DNA nanotechnology to (self-)assemble DNA origami [36–39], DNA superlattices [40], Borromean rings [41] and complex-shaped nanoscale objects [42], such as gears, stars and smileys. At a higher level of genetic material organization, we find that the three-dimensional architecture of the 30 nm chromatin fiber is largely affected by the formation of loops [43, 44]. These structures are stabilized by protein complexes, e.g. the CTCF transcription factor, selectively bridging specific binding sites along the DNA sequence [45–47]. The formation of these loops plays a crucial role in the regulation of gene expression.

Natural knots in the genetic material remain nonetheless elusive. As previously noted, in fact, a survey of RNA molecules indicated a remarkable absence of knotted structures [24], in spite of the capability, in principle, to exploit secondary structure formation to achieve topologically entangled folds akin to those observed in knotted proteins. Similarly, in the case of nuclear chromatin the length of the fiber (which largely exceeds that of the CTCF-induced loops), the activity of topology-regulating enzymes such as topoisomerases [48–50], and non-equilibrium dynamics [23] make it impossible for these loops to become elements of topological entanglement, i.e. to knot the chromosome. However, the mechanisms underlying loop formation in both aforementioned cases are completely general, and, if properly designed and applied to shorter fibers, could be exploited to manipulate their knotted state.

**Fig 1:**
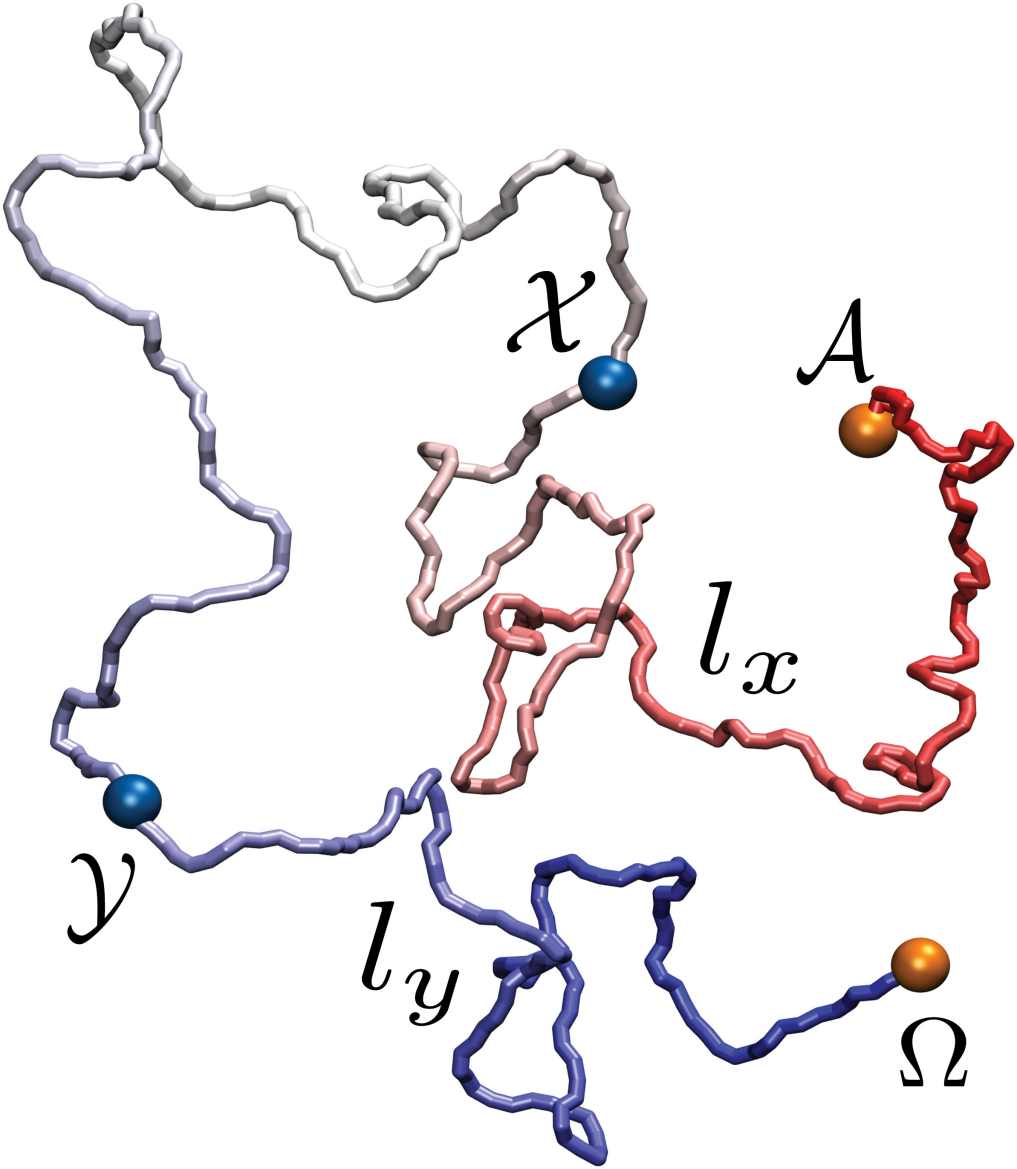
Snapshot of the DNA model under exam. The orange beads, labeled 𝓐 and Ω, indicate the sticky patches at the termini of the polymer, while the blue beads, labeled 𝓧 and 𝓨, represent those along the polymer chain. These two pairs of regions do not attract each other. The chain segment comprised between 𝓐 and 𝓧 (resp. 𝓨 and Ω) has length *l*_*x*_ (resp. *l*_*y*_).

Here we investigate what impact the formation of a loop can have on the topology of a DNA fiber. Specifically, we consider a filament of double-stranded DNA (dsDNA) modeled as a chain of beads with excluded volume [7, 12, 23, 51], and introduce two pairs of adhesive monomers, as illustrated in Fig 1, which permanently stick to each other when sufficiently close. One pair of such monomers (labeled 𝓐, Ω) is located at the termini, and has the role of circularizing the polymer to freeze its topological state. A second pair of adhesive monomers (labeled 𝓧, 𝓨), not interacting with the first, is located along the chain. The latter is initially set up in an open, linear conformation. A constant-temperature molecular dynamics (MD) simulation is carried out until the termini become close enough to stick and cyclize the polymer. The knotted state of the resulting conformation is then analyzed as a function of the position of the 𝓧, 𝓨 sticky regions. Two types of DNA models, termed L-DNA and S-DNA, are employed, both composed by polymer chains having the same number of monomers but different persistence length. The first case models a dsDNA chain long enough so that its persistence length is negligible; the second case corresponds, for dsDNA at physiological salt conditions (0.15 M NaCl) [52], to a 7.5 kbp long filament, roughly the length of the papillomavirus genome [53]. Further details on the model and the simulation protocol are reported in the Material and Methods section.

## Results and discussion

**Fig 2:**
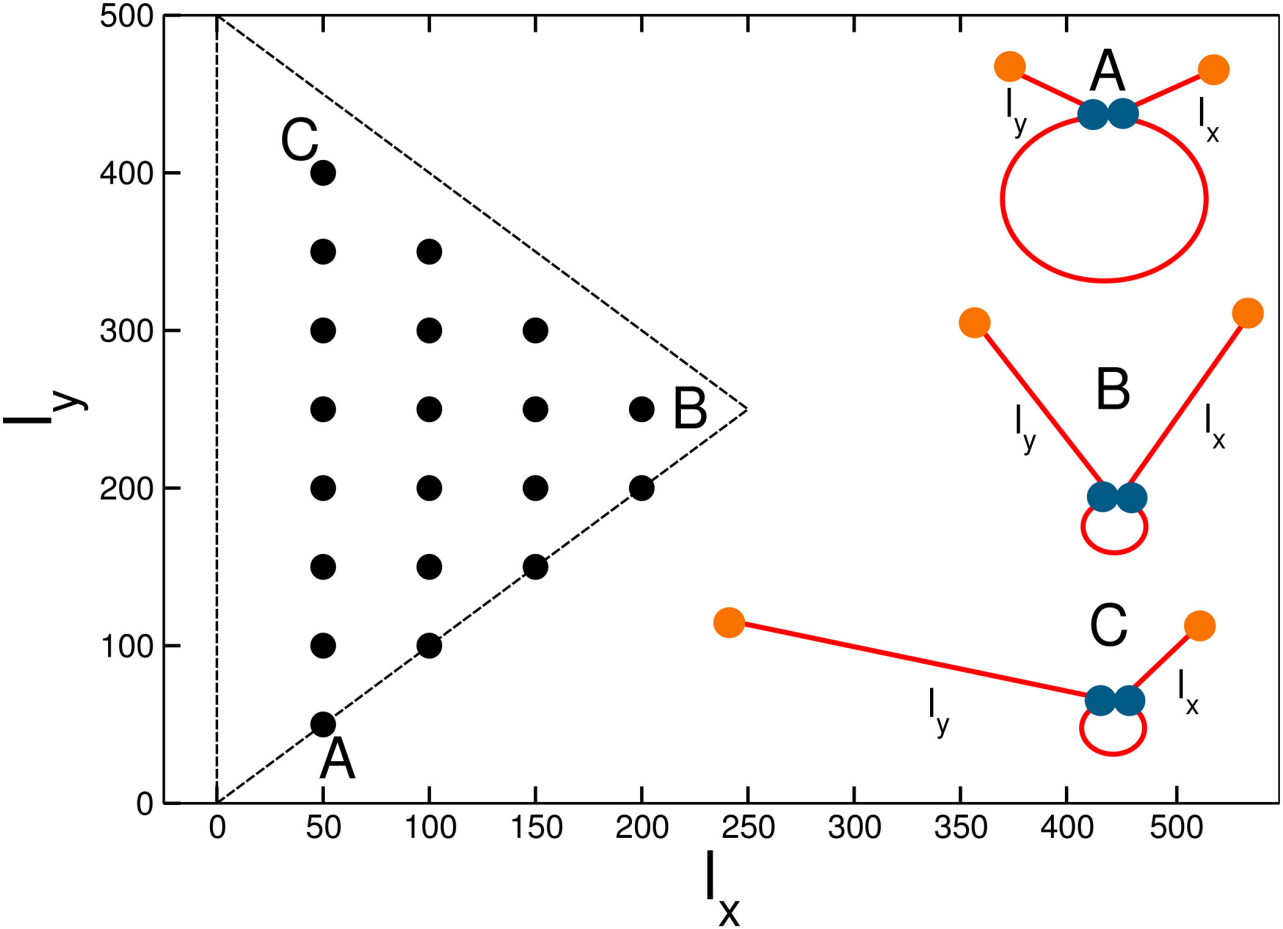
The parameter space explored in the present study. The coordinate on each axis represents the length of an *arm*, i.e. the number of polymer beads between a terminus and the closest sticky bead along the sequence. The dots indicate the values that have been investigated. In the right half of the plot, three sketches of the polymer with closed sticky loop illustrate the A, B, C points.

The sticky monomers 𝓧, 𝓨, located along the chain, identify two types of subchains: the *arms* of the polymer, namely the chain segments between a terminus and the closest sticky bead along the sequence ([𝓐 − 𝓧] and [Ω − 𝓨]); and the *sticky loop*, i.e. the segment comprised between the central sticky beads ([𝓧 − 𝓨]). The chains are composed by *N* = 500 beads, along which the sticky monomers can be located only at distances *l*_*x*_ = 50*n*_*x*_, *l*_*y*_ = 500 − 50*n*_*y*_ from the termini, with *n*_*x*_, *n*_*y*_ = 1, 2, ⋯ 9. Of the 9 × 9 = 81 possible configurations we have to exclude the ones in which the beads coincide and, for symmetry, the pairs in which $l_{x}=l_{y}^{\prime },l_{y}=l_{x}^{\prime }$. This leaves us with 20 non-redundant locations of the sticky bead pairs along the chain. In Fig 2 the configurations are distributed in the *l*_*x*_, *l*_*y*_ plane; along the positive-tangent diagonals (parallel to the A–B line) one has configurations in which the length of the arms changes but it is the same for the two arms, while along the negative-tangent diagonals (parallel to the C–B line) the size of the loop stays constant, but its location along the chain changes. An illustration of the three extreme cases is also provided.

For each of the 20 locations of the adhesive monomers we measured the relative knotting probability (RKP) of the polymer, defined as:

$$\text{RKP}=\frac{P_{K}\left(l_{x},l_{y}\right)}{P_{K}^{0}}$$ (1)

where *P*_*K*_(*l*_*x*_, *l*_*y*_) is the knotting probability of the chain with both types of sticky monomers, and $P_{K}^{0}$ is the (reference) knotting probability of an equivalent chain with adhesive termini only. These probabilities are computed as the fraction of knotted final configurations over the total. For the two types of chain under exam, namely the L-DNA and the S-DNA, we performed 104 ⋅ 10^3^ and 24 ⋅ 10^3^ independent simulations, respectively; as discussed in the Materials and Methods section, the larger number of runs for the L-DNA with respect to the S-DNA is required by the smaller knotting probability of the former over the latter [54].

As anticipated in the Introduction, the final configuration is defined as the last frame of a MD simulation; the latter is interrupted when the terminal sticky beads cyclize the chain, irrespective of whether the 𝓧, 𝓨 sticky monomers are joint or not. It has to be stressed that the presence of the adhesive termini restricts the conformational space the polymers can sample. The circularization of the chain, in fact, freezes the topology and prevents the polymer from changing its knotted state, either by tying a more complex knot or by untying the existing one. This constraint, however, reduces the complexity of the phenomenology under exam and removes any possible source of ambiguity due to the detection of the knotted state of an open chain [55].

The scheme in Fig 2 allows us to predict the qualitative behavior of the RKP as a function of the sticky monomer locations. First, we expect to observe an overall increase of the knotting probability with respect to a chain without central adhesive monomers: these, in fact, favor more dense configurations, thus “self-confining” the polymer and enhancing its propensity to entangle [56]. Second, in the configurations corresponding to the corners of the triangle the sticky beads are very close to the termini (point A in Fig 2) or to each other (point B) or both (point C). Therefore, their effect on the topology will be negligible, and the RKP should be almost unity in those points. Along the A-B and A-C segments the size and position of the sticky loop can vary. For Rolle’s theorem [57], then, we expect the presence, along these sides of the triangle, of a local maximum of the probability. The case for the C-B side is different: along the latter, in fact, the location of the sticky beads changes, but the size of the loop remains the same—zero in the limiting case in which the two sticky monomers coincide. Hence, the RKP should remain fixed at unity.

**Fig 3:**
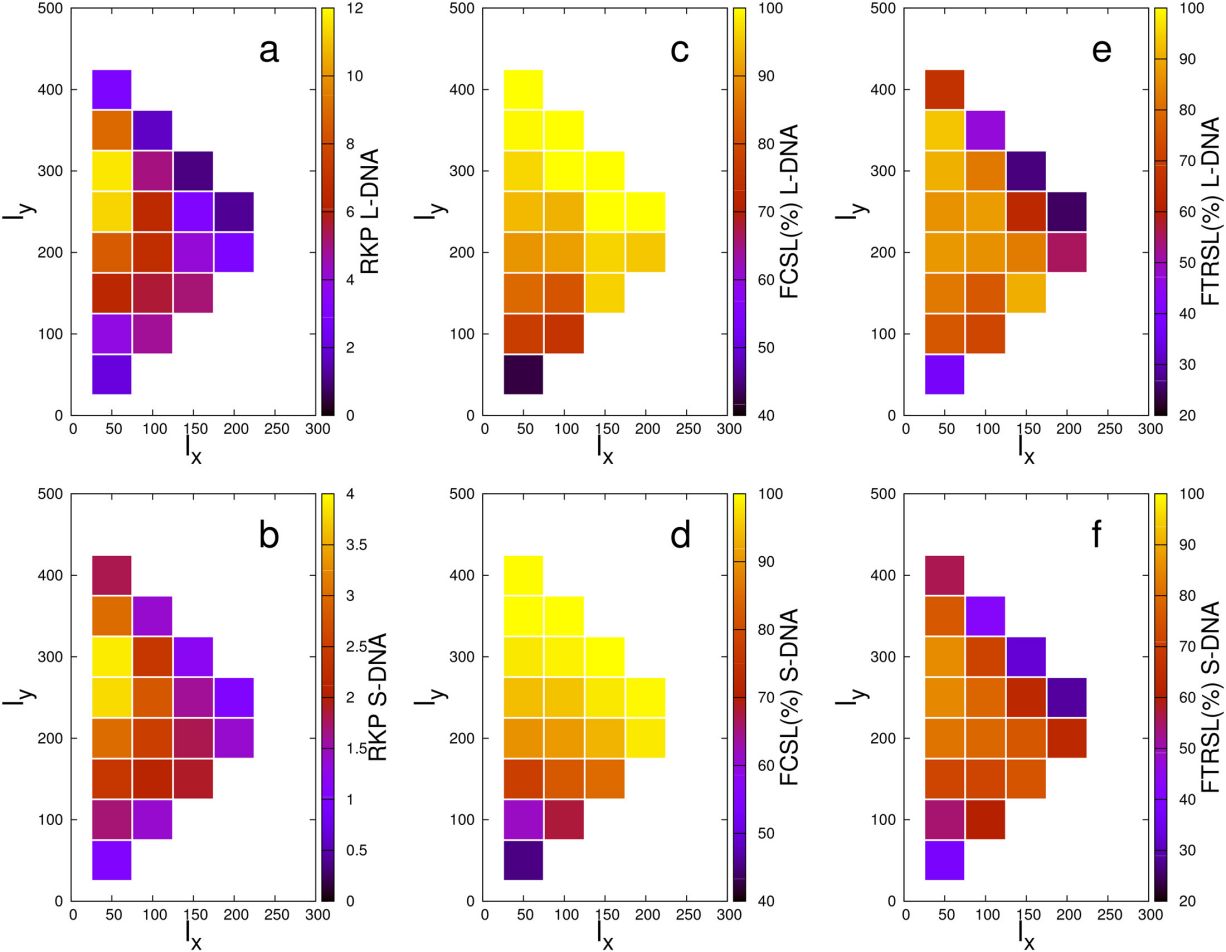
Heat-map representation of the simulation data as a function of the sticky monomer location. Top row: data for the L-DNA. Bottom row: data for the S-DNA. RKP: relative knotting probability. FCSL: fraction of knotted chains with closed sticky loop. FTRSL: fraction of knotted chains with topologically relevant sticky loops.

The predicted behavior is confirmed by the heat-map plots shown in Fig 3a and 3b, which report the RKP for L-DNA and S-DNA, respectively. As expected, for all points we have RKP ≥ 1, which indicates an overall enhancement of the knotting propensity in presence of the sticky monomers. The maximum relative increase amounts to 11.6 for the L-DNA and 3.9 for the S-DNA.

A remarkable result is that, in spite of a noticeable difference in the absolute numbers, the qualitative behaviors of the RKPs of L-DNA and S-DNA are decidedly similar. Not only, in fact, both probability distributions comply with the expected presence of maxima along the A-B and A-C segments; additionally, the absolute maximum of the RKP occurs in the same point of the plane, namely the {50, 300} point, for both chain types. The fact that this particular pair of arm lengths maximizes the knotting probability results from the interplay of two competing effects: on the one hand, the size of the loop has to be sufficiently large in order to allow and favor the threading of the arms through it; on the other hand, an adequate length difference between the arms has to be guaranteed, so that one of them is short enough to pierce through the loop before the terminal sticky monomers come close together and freeze the topology in an unknotted state. As a matter of fact, in this configuration the 𝓧, 𝓨 sticky beads are only 150 monomers apart, so that they can “find” each other before the termini do the same. At the same time, the loop is large enough to allow either arm to go though it (if it were perfectly circular, its diameter would be roughly 50 beads). Consistently with these observation, we also note that the relative maximum of the RKP along the A-B line (that is, for equal length of the arms) is located at the {150, 150} point: in this configuration, the sticky loop is 200 beads long, very close to the optimal length.

As already noted, the location of the maxima is the same in the two cases under exam, irrespectively of the persistence length of the chain. If we assume the same fine-grained system to underlie both models, we can rephrase this observation by saying that DNA chains of different length feature the same optimal location of the sticky patches. From this perspective, we thus understand the optimization of the knotting probability by means of the introduction of adhesive regions as a property that only depends on their position relative to the chain length, with the latter only affecting the absolute value of the enhancement of the knotting probability.

**Fig 4:**
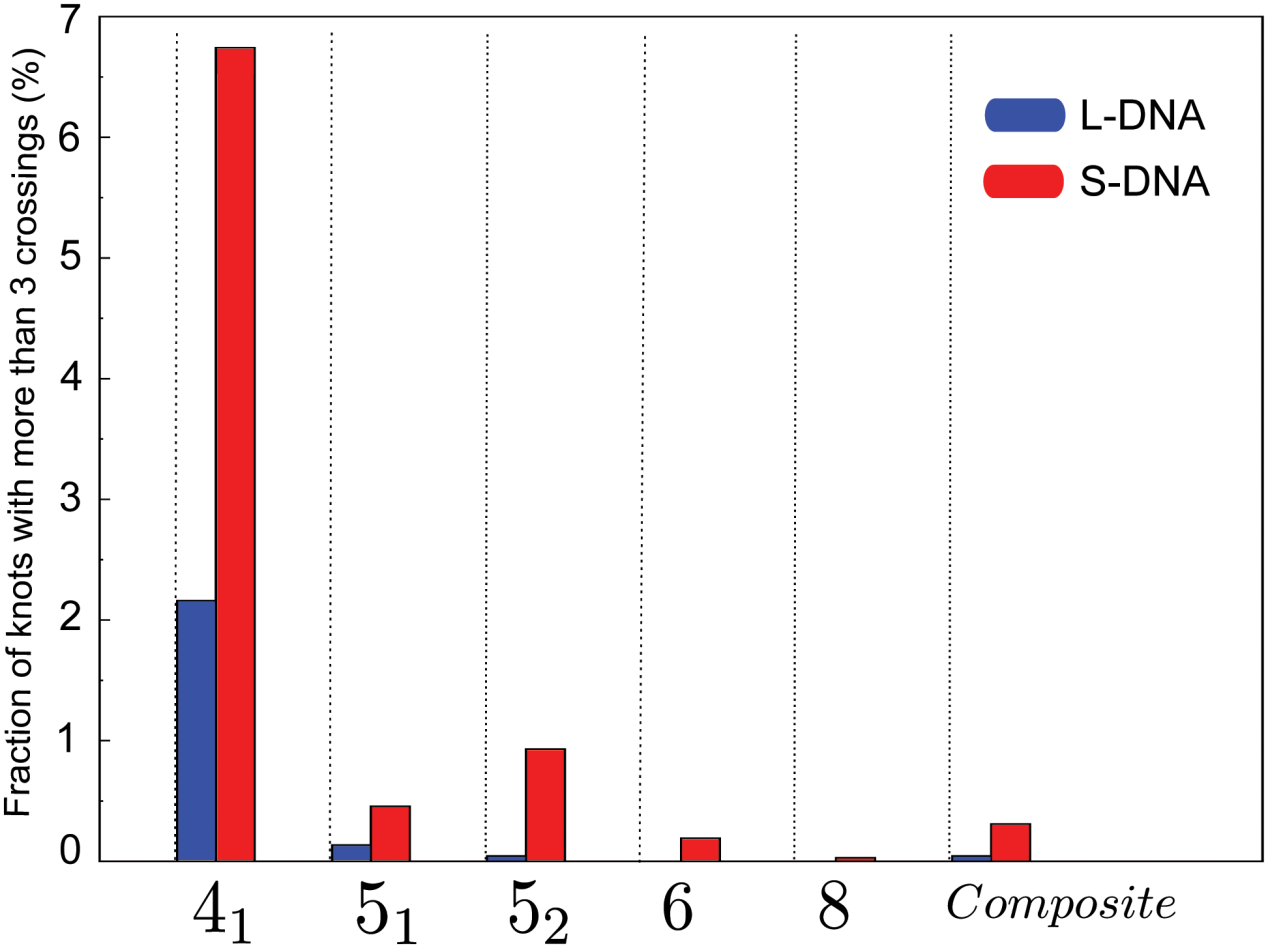
Cumulative knot spectrum of the L-DNA (blue) and S-DNA (red) chains, irrespective of the sticky monomer location. The percentage refers to the total number of knotted configurations. The fraction of 3_1_ knots is ∼ 98% and ∼ 91.5% for L-DNA and S-DNA, respectively.

Further insight comes from the analysis of the knot complexity in the two cases under exam (numerical values are reported in S1 Table). In fact, the vast majority (> 98% on average) of the knots observed in the L-DNA are 3_1_, while in the S-DNA case the fraction of knots more complex than a 3_1_ can be larger than 8.4%. A possible explanation for this tendency towards complex knots in the S-DNA is that some degree of bending rigidity allows for larger, more open loops that favor multiple threading. More detail is provided in Fig 4, where we report the knot spectrum for the two chain types cumulated over all possible sticky monomer location. (The break-down of the spectrum for all 20 sticky monomer locations under exam is provided in Fig B in S1 Text) From the S-DNA data we see that the 5_2_ knot type is more abundant than the 5_1_ by approximately a factor 2. The same trend is observed [11] in simulations of DNA under confinement. The knotting mechanism in the aforementioned condition cannot, obviously, be compared with the knotting experienced by the chains under exam in the present work. It is however interesting to observe how the same behavior can emerge by means of the *self-confinement* introduced by the formation of the sticky loop. In the L-DNA case we observe the opposite balance between 5_1_ and 5_2_ knots, but their abundance is too small to rule out an insufficient sampling.

The data reported in Fig 3a and 3b show that, in general, the presence of the sticky monomers along the chain increases the relative knotting probability with respect to equivalent chains having only the sticky termini. This observation alone, however, does not provide any information about the role played by the formation of a sticky loop. To ascertain this, we measured how many knotted configurations involve the latter. These values are graphically illustrated in Fig 3c and 3d for the L-DNA and S-DNA, respectively. The fraction of knotted chains in which the loop is closed ranges between 84% and 100% for 31 of the 40 cases under exam, depending on the sticky monomer location. More specifically, and not surprisingly, in both L-DNA and S-DNA we observe a positive gradient in the fraction of closed loops in the direction of shorter distances between the 𝓧, 𝓨 beads. In other words, the closer the sticky beads are along the sequence, the higher the probability that they will adhere. For the sticky bead location maximizing the RKP, namely the {50, 300} point, the fraction of knotted configurations involving a closed sticky loop amounts to 96.6% for L-DNA and 98.1% for S-DNA.

**Fig 5:**
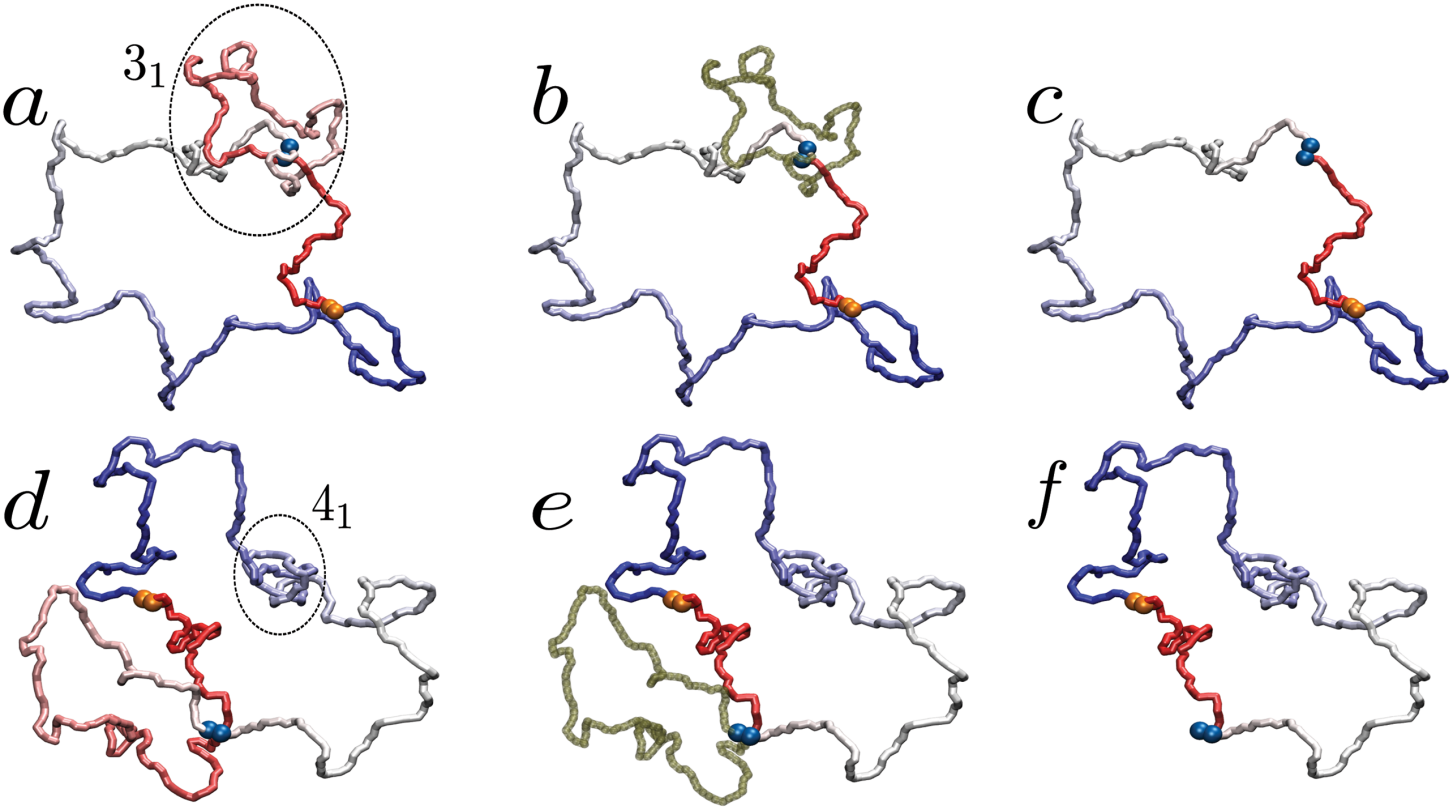
Illustration of the algorithm employed to determine whether a sticky loop is relevant or not for the topological state of the polymer. If the sticky loop (dark green segment) is entangled with the knot (panels a and b), its removal determines a change in the chain topology (panel c). On the contrary, if no chain segment pierces the sticky loop (panels d and e), its excision does not modify the topological state of the whole polymer (panel f). In the figure, the sticky beads at the termini are orange, while those along the sequence are blue. In the first panel of both cases (a and d) the knotted region is inscribed in a circle. The two configurations are obtained from simulations of S-DNA with sticky monomers located at the {50, 300} point.

Having assessed that the sticky loops are present in most of knotted configurations, we need to discriminate between the case in which the loop and the knot coexist without interfering and the case in which loop and knot are topologically entangled. These two possibilities are depicted in Fig 5. Given a closed, knotted chain featuring a sticky loop, we deem the latter to be *topologically relevant* if its removal determines a change in the topology of the chain (panels a to c). Alternatively, we consider the sticky loop irrelevant to the formation of the knot if the latter is completely localized within the sticky loop or in the complementary loop obtained by removing the former (as illustrated in panels d to f). A more detailed discussion of this algorithm is provided in section C in S1 Text.

As shown in Fig 3e and 3f, the amount of loop-dependent knots is in general large among the different sticky beads locations, half of them having a percentage equal to or larger than 75%. Also much smaller values are present in some cases, though never below 25%. The highest values are registered in proximity of the RKP {50, 300} maximum, namely 90.7% for L-DNA and 85.4% for S-DNA, thus suggesting that the formation of a stable loop in the appropriate region of the chain indeed enhances the probability to form a knot by threading a polymer terminus through it.

## Conclusions

In summary, we have demonstrated that the knotting probability of a filament of dsDNA, modeled as a thick self-avoiding chain, can be increased by introducing, along its sequence, two adhesive regions. The extent of this enhancement depends only on the location of the latter, and its qualitative pattern is the same for the two chain types considered, namely with zero and finite persistence length.

The highest chance of self-entangling the polymer is obtained when the location of the adhesive regions optimizes the interplay of two competing effects: one is the enhancement of the threading probability, which is proportional to the loop size; the other is the polymer circularization event, which is more probable when the length difference between the two arms is small. Our data indicate that the knotting probability is maximized, in both L-DNA and S-DNA, when the sticky monomers allow the formation of a loop of length ∼ 1/3 of the whole polymer located close to one of the termini.

Because of the generality of the model employed in this study, the presented results are prone to verification and employment on a fairly broad range of length scales: in fact, as mentioned in the Introduction, genetic material at different length scales -from single-stranded RNA to 30 nm chromatin fibers- is provided with the elements necessary to form loops. The knowledge of the mechanisms favoring the realization of knotted topologies can, therefore, be employed not only to design self-knotting structures, but also to rationalize the absence of entanglement in otherwise knot-prone systems.

Understanding how the sequence of a polymer determines not only its geometrical structure but also its knotted state is a relevant and difficult task. The model discussed here aimed at reducing the complexity of the problem to the core by introducing the smallest amount of sequence information in a plain polymer with excluded volume. The rich behavior featured by this simple system proved useful to build a basic understanding of the relation between the constituents of a complex molecule and its topological state, and provided the instrument for future work that will elucidate the knotting process.

## Materials and Methods

### Model and simulation details

For our study we employed the well-established Kremer-Grest model of a coarse-grained polymer [58]. Specifically, our polymer chain is modeled as a collection of identical beads of unit mass, connected by anharmonic FENE bonds [58]. The non-bonded interaction acting among the beads is a Weeks-Chandler-Anderson [59] (WCA) potential, which enforces the excluded volume. The only beads featuring a further non-bonded potential are the termini and the two sticky beads along the chain. The most general form of the total potential energy of the chain is:

$$\begin{array}{c} 𝓗=U_{WCA}+U_{FENE}+U_{stick}+U_{bend} \end{array}$$ (2)

The WCA potential is given by:

$$\begin{array}{ccc} & & U_{WCA}=\frac{1}{2}\sum_{(i,j),j\neq i}^{N}V\left(d_{i,j}\right), \end{array}$$ (3)

$$V\left(r\right)=\{\begin{array}{c} 4\epsilon \;\left[{\left(\frac{\sigma }{r}\right)}^{12}-{\left(\frac{\sigma }{r}\right)}^{6}+\frac{1}{4}\right]\;\text{for}\;r\leq 2^{1/6}\sigma \\ 0\;\;\text{otherwise} \end{array}$$ (4)

where *ϵ* = 1 sets the energy scale. The FENE potential reads:

$$U_{FENE}=-\sum_{i=1}^{N-1}\;\frac{\kappa_{fene}}{2}\;{\left(\frac{R_{0}}{\sigma }\right)}^{2}\text{ln}\;\left[1-{\left(\frac{d_{i,i+1}}{R_{0}}\right)}^{2}\right]$$ (5)

where $d_{i,i+1}=|{\vec{r}}_{i}-{\vec{r}}_{i+1}|$ is the distance of the bead centers *i* and *i* + 1, *R*_0_ = 1.5*σ* is the maximum bond length and *κ*_*fene*_ = 30*ϵ* is the interaction strength.

The sticky interaction is modeled as a negative Gaussian short range potential acting between sticky beads of like type. The potential is given by:

$$\begin{array}{ccc} & & U_{stick}=G(|{\vec{r}}_{\Omega }-{\vec{r}}_{𝓐}\left|\right)+G(|{\vec{r}}_{𝓨}-{\vec{r}}_{𝓧}\left|\right)\\ & & G\left(r\right)=\{\begin{array}{c} -U_{0}\exp(-\frac{{\left(r-2^{1/6}\sigma \right)}^{2}}{2\lambda^{2}})\;\text{for}\;2^{1/6}\sigma \leq r\leq \left(2^{1/6}+5\right)\sigma \\ 0\;\;\text{otherwise} \end{array} \end{array}$$ (6)

where ${\vec{r}}_{𝓐}$, ${\vec{r}}_{\Omega }$, ${\vec{r}}_{𝓧}$ and ${\vec{r}}_{𝓨}$ are the coordinates of sticky beads 𝓐, Ω, 𝓧 and 𝓨, respectively. The parameters *U*_0_ = 100*ϵ* and *λ* = 2.5*σ* are chosen so that the interaction between sticky monomers is sufficiently strong, i.e. larger than thermal fluctuations, when they are closer than 2 − 3*σ*.

The bending rigidity potential is defined as:

$$\begin{array}{c} U_{bend}=\sum_{i=1}^{N-2}\frac{\kappa_{bend}}{2}\;{\left(\theta_{i}-\pi \right)}^{2} \end{array}$$ (7)

where *θ*_*i*_ is the angle formed by a triplet of consecutive beads with the *ith* bead at the center. The bending stiffness is *κ*_*bend*_ = 10*k*_*B*_ *T*, with *k*_*B*_ Boltzmann constant.

The constant-temperature MD simulations are carried out with an in-house code integrating the Langevin equations of motion with *k*_*B*_ *T* = *ϵ* and $\tau =\sigma \sqrt{m/\epsilon }=1$ MD time units. In the L-DNA case (resp. the S-DNA case), a total number of 104 ⋅ 10^3^ (resp. 24 ⋅ 10^3^) simulations have been performed for each of the 20 central sticky monomer locations. The factor ∼ 4 separating the numbers of individual runs of the two sets depends on the different knotting probability between L-DNA and S-DNA, respectively 1.9417 ⋅ 10^−4^ and 6.6680 ⋅ 10^−3^. Since the latter is much higher than the former (as discussed e.g. in [54]) we performed a substantially larger number of runs for the L-DNA case in order to obtain a statistically significant number of knotted conformations for both polymer chain types.

### Knot analysis

Knots are mathematically well-defined only for closed curves. As our simulation protocol automatically returns circularized conformations, we are spared from the need to perform a chain closure [55], a time-consuming and potentially ambiguous procedure (especially in the case of buried termini). The topological state of our closed chains has been obtained applying the algorithm implemented in the KNOTFIND [60] package.

## Supporting Information

S1 Text Computational and result details. Computation of the persistence length; Simulation protocol; Knot analysis; Knot spectrum for all sticky bead positions.
(PDF)

S1 Table Result details. Numerical values of the results of the present work.
(PDF)
