# Supplementary material for: Two Adhesive Sites Can Enhance the Knotting Probability of DNA
Source: PLoS One. 2015 Jul 2;10(7):e0132132. doi: 10.1371/journal.pone.0132132 (PMC4489926; doi:10.1371/journal.pone.0132132)
Supplement: S1 Text — Computation of the persistence length; Simulation protocol; Knot analysis; Knot spectrum for all sticky bead positions. (PDF) [file pone.0132132.s001.pdf]

Supporting Information for

Two adhesive sites can enhance the knotting probability of DNA

Saeed Najafi and Raffaello Potestio
Max Planck Institute for Polymer Research, Ackermannweg 10, 55128 Mainz, Germany

A. COMPUTATION OF THE PERSISTENCE LENGTH

The system under study is a 500 bead long polymer. The diameter σ of the beads is taken as a length unit, and the same distance separates the centers of consecutive beads. Because of the short-range WCA [1] repulsion, the chain cannot cross itself. Two types of polymer are employed in this work, namely one in which no bending rigidity is present (the L-DNA), and one in which a three-body bonded potential imposes a given stiffness. These two types of chain thus feature different persistence length l_P, that is, the characteristic decay length of the bond vector self correlation [2].

In the first case the expression for l_P is given by [3]:

$$l_P = -\frac{\sigma}{\ln(1 - \frac{\sigma^2}{4\Delta^2})} \tag{1}$$

where σ is the bond length and Δ is the cross-sectional radius of the chain. In the case under exam, we have $\Delta \simeq \sigma/2$: with this value in Eq. 1 we obtain $l_P = 0$.

For the case in which a bending rigidity κ_{bend} is present (S-DNA), we impose its strength based on the requirement to reproduce DNA in physiological salt conditions, i.e. 0.15 M NaCl. Following Ref. [4], we have that in this case the effective diameter of DNA is $\sigma = 5nm$. Assuming a persistence length of 50 nm or 150 bp we obtain the appropriate bending energy for this condition as [4]:

$$\frac{\kappa_{bend}}{k_B T} \simeq \frac{l_P}{\sigma} = 10 \tag{2}$$

For a polymer of length $L = 500\sigma$ as in our case, we obtain $L/l_P = 50$, corresponding to $150bp \times L/l_P = 7500bp$.

B. SIMULATION PROTOCOL

In order to investigate how the presence and location of the sticky beads along the polymer chain affect the polymer topology we performed different sets of simulations. Each of these corresponds to a particular location of the central sticky beads. Specifically, we placed the sticky monomers in all possible, non-redundant locations separated by 50 beads available on a chain of polymerization degree $N = 500$. This choice leaves us with 20 possible locations.

For each position of the central sticky monomers, two different blocks of simulations are performed, one for L-DNA and one for S-DNA. A single simulation runs according to the following protocol:

1. the polymer chain is initialized in a randomized, extended conformation

2. the Langevin equations of motion are integrated

3. as soon as the two terminal beads "stick together" through the Gaussian potential the simulation is interrupted - whether the central sticky beads have adhered to each other or not

4. the final configuration of the polymer chain is stored; the trajectory is discarded

C. KNOT ANALYSIS

The topological state of our closed chains has been obtained applying the `KNOTFIND` algorithm [5].

In our analysis, we had to take into account the fact that the overall topological state of the chain might be independent of the loop formed by the central sticky monomers. In order to ascertain the entanglement between knot and sticky loop in a knotted configuration, we considered all those chains that have a nontrivial topology *and* a sticky loop, i.e., the central sticky beads $\{\mathcal{X}, \mathcal{Y}\}$ have adhered before the termini did. We then identify all monomers in the sticky loop (that is, all monomers having index $i \in (\mathcal{X}, \mathcal{Y})$) and the complementary loop formed by the two arms, and analyze their topology. If one of them is in the same knotted state of the full chain, we deduce that the knot has formed deep into the loop but not because of it, or equivalently on the arms. On the other hand, if both the sticky loop and the loop formed by the two arms alone have a different topology with respect to the full chain, the loop is deemed to be topologically relevant.

It is worth stressing here the the sticky loop can result to be topologically relevant whether it was necessary to the formation of the knot or not. The closure of the loop, in fact, can occur *after* the knotting of the chain and entangle it *a posteriori*, so that the loop removal would result in a crossing, e.g. as illustrated in Fig. A. Our analysis, which is based on the sole final configuration, does not explicitly identify these cases, rather it provides

a measure of the overall topological and geometrical entanglement in the polymer.

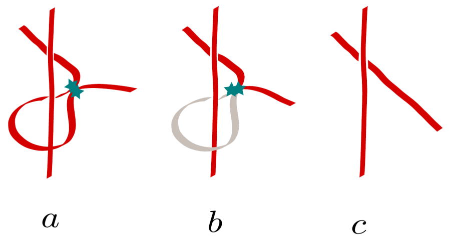

FIG. A: Illustration of the process by which a the excision of a sticky loop results in the swapping of two chain segments. For this to happen, the sticky loop has to be pierced by another stretch of the chain.

D. KNOT SPECTRUM FOR ALL STICKY BEAD POSITIONS

We report in Fig. B hereafter the break-down of the knot spectrum for the two DNA types, separated by sticky bead position. The $0-0$ label indicates the reference chains without central $(\mathcal{X}, \mathcal{Y})$ sticky beads.

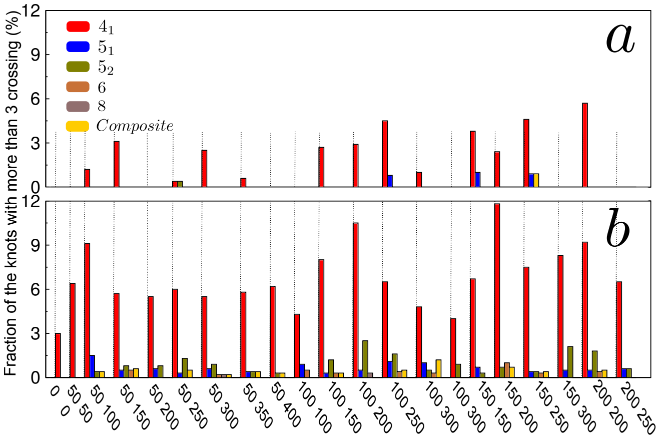

FIG. B: Knot spectrum of L-DNA (panel a) and S-DNA (panel b). The $0-0$ label indicates the reference simulations where only the terminal sticky monomers are present.

[1] J. D. Weeks, D. Chandler, and H. C. Andersen, J. Chem. Phys. **54**, 5237 (1971).

[2] P. Flory, *Statistical Mechanics of Chain Molecules* (Hanser, 1969).

[3] C. Micheletti, D. Marenduzzo, and E. Orlandini, Physics Reports **504**, 1 (2011).

[4] V. V. Rybenkov, N. R. Cozzarelli, and A. V. Vologodskii, Proceedings of the National Academy of Sciences **90**, 5307 (1993).

[5] J. Hoste and M. Thistlethwaite, KNOTFIND, 1999, www.math.utk.edu/morwen/knotscape.html.
