# Supplementary material for: Two Adhesive Sites Can Enhance the Knotting Probability of DNA
Source: PLoS One. 2015 Jul 2;10(7):e0132132. doi: 10.1371/journal.pone.0132132 (PMC4489926; doi:10.1371/journal.pone.0132132)
Supplement: S1 Table — Numerical values of the results of the present work. (PDF) [file pone.0132132.s002.pdf]

		RKP		Knots with more than 3 crossings (%)		FCSL (%)		FTRSL (%)	
**N**	l_x l_y	**L-DNA**	**S-DNA**	**L-DNA**	**S-DNA**	**L-DNA**	**S-DNA**	**L-DNA**	**S-DNA**
**1**	50 50	2.0	1.0	—	4_1 (6.4)	42.5	45.2	37.5	38.2
**2**	50 100	3.9	1.7	4_1 (1.2)	4_1 (9.1), 5_1 (1.5), 5_2 (0.4), $3_1\#3_1$ (0.4)	77.2	61.7	75.9	54.2
**3**	50 150	6.4	2.4	4_1 (3.1)	4_1 (5.7), 5_1 (0.5), 5_2 (0.8), 6_1 (0.5), $3_1\#3_1$ (0.6)	84.5	77.6	82.2	71.7
**4**	50 200	8.5	3.0	—	4_1 (5.5), 5_1 (0.6), 5_2 (0.8)	90.2	89.8	87.3	81.6
**5**	50 250	11.3	3.8	4_1 (0.4), 5_2 (0.4)	4_1 (6.0), 5_1 (0.3), 5_2 (1.3), $3_1\#3_1$ (0.5)	93.9	94.3	86.5	85.1
**6**	50 300	11.6	3.9	4_1 (2.5)	4_1 (5.5), 5_1 (0.6), 5_2 (0.9), $3_1\#3_1$ (0.2), 6_3 (0.2), 8_{21} (0.2)	96.6	98.1	90.7	85.4
**7**	50 350	8.9	3.0	4_1 (0.6)	4_1 (5.8), 5_1 (0.4), 5_2 (0.4), $3_1\#4_1$ (0.4)	99.4	99.8	93.4	76.2
**8**	50 400	2.9	1.8	—	4_1 (6.2), 5_2 (0.3), $3_1\#3_1$ (0.3)	100	99.7	66.1	55.9
**9**	100 100	4.8	1.4	—	4_1 (4.3), 5_1 (0.9), 6_1 (0.5)	75.3	67.8	72.1	60.4
**10**	100 150	5.6	2.1	4_1 (2.7)	4_1 (8.0), 5_1 (0.3), 5_2 (1.2), $3_1\#3_1$ (0.3), 6_3 (0.3)	81.4	82.2	76.1	71.8
**11**	100 200	6.8	2.5	4_1 (2.9)	4_1 (10.5), 5_1 (0.5), 5_2 (2.5), 8_1 (0.3)	91.4	90.7	86.3	79.2
**12**	100 250	6.6	2.8	4_1 (4.5), 5_1 (0.8)	4_1 (6.5), 5_1 (1.1), 5_2 (1.6), 6_1 (0.2), $3_1\#3_1$ (0.5), 6_2 (0.2)	93.2	94.9	88.0	79.0
**13**	100 300	5.0	2.4	4_1 (1.0)	4_1 (4.8), 5_1 (1.0), 5_2 (0.5), $3_1\#3_1$ (0.8), $4_1\#4_1$ (0.3), 6_2 (0.3)	100	99.0	82.2	71.3
**14**	100 350	1.7	1.4	—	4_1 (4.0), 5_2 (0.9)	100	100	47.1	42.2
**15**	150 150	5.1	1.9	4_1 (3.8), 5_1 (1.0)	4_1 (6.7), 5_1 (0.7), 5_2 (0.3)	96.2	84.9	90.4	75.3
**16**	150 200	4.1	1.8	4_1 (2.4)	4_1 (11.8), 5_2 (0.7), 6_1 (0.7), $3_1\#3_1$ (0.7), 6_3 (0.3)	96.3	93.4	82.9	75.8
**17**	150 250	3.0	1.6	4_1 (4.6), 5_1 (0.9), $3_1\#5_2$ (0.9)	4_1 (7.5), 5_1 (0.4), 5_2 (0.4), 6_1 (0.3), $3_1\#3_1$ (0.4)	100	98.1	63.3	63.7
**18**	150 300	1.0	1.2	—	4_1 (8.3), 5_1 (0.5), 5_2 (2.1)	100	100	26.5	32.6
**19**	200 200	2.9	1.4	4_1 (5.7)	4_1 (9.2), 5_1 (0.5), 5_2 (1.8), 6_1 (0.4), $3_1\#3_1$ (0.5)	95.2	98.2	55.2	63.6
**20**	200 250	1.2	1.0	—	4_1 (6.5), 5_1 (0.6), 5_2 (0.6)	100	99.4	25.0	28.6

TABLE I. Summary of the data presented in the present work. RKP: relative knotting probability. FCSL: fraction of knotted chains with closed sticky loop. FTRSL: fraction of knotted chains with topologically relevant sticky loops.
